# Comparison of Self-Reported Substance Use with Outcomes of Urine Testing among Men Who Have Sex with Men in Africa Participating in HPTN 075

**DOI:** 10.1007/s10461-025-04832-6

**Published:** 2025-09-13

**Authors:** Theodorus G. M. Sandfort, Susan H. Eshleman, Justin Knox, Autumn Breaud, Katie Weaver, Emily Kerubo, Ravindre Panchia, erica l. hamilton, Vanessa Cummings, Bill Clarke

**Affiliations:** 1 Department of Psychiatry, HIV Center for Clinical and Behavioral Studies, New York State Psychiatric Institute and Columbia University, 1051 Riverside Dive, Unit 15, New York, NY 10032 USA; 2 Department of Pathology, Johns Hopkins University School of Medicine, Baltimore, MD USA; 3 Department of Sociomedical Sciences, Columbia University, New York, NY USA; 4 Kenya Medical Research Institute (KEMRI) CDC, Kisumu, Kenya; 5 Perinatal HIV Research Unit, University of the Witwatersrand, Soweto, South Africa; 6 Science Facilitation Department, Durham, NC FHI 360 USA

**Keywords:** Validity self-report, Substance use, Urine testing, Men who have sex with men, Sub-Saharan Africa

## Abstract

Research linking substance use with HIV risk behaviors and antiretroviral therapy adherence is typically based on self-reported data. However, studies in high-income countries show that men who have sex with men (MSM) often underreport substance use when compared to objective biological testing. Such comparisons have not been conducted among MSM in sub-Saharan Africa. We compared self-reported and objectively measured substance use among MSM participating in HPTN 075, a multi-site observational cohort study conducted in Kenya, Malawi, and South Africa. Urine samples (*n* = 734) from 382 participants were tested for the alcohol metabolite ethyl glucuronide and 43 other substances. These tests detect alcohol use in the prior 48–72 h and other substances up to 7 days earlier, depending on the drug. Of the 734 samples, 159 (21.7%) tested positive for ethyl glucuronide. Self-reported alcohol use was available for 97.5% of these cases and confirmed in 141 (91.0%) of them. Sixty samples (8.2%) tested positive for at least one of the 43 other substances. Self-report data were available for 95.0% of those, but use was acknowledged in only 19 (33.3%) cases. These findings suggest that alcohol use is generally reported accurately, while drug use is substantially underreported—likely due to legal prohibitions and social stigma. Incorporating objective substance testing alongside self-reports is recommended to improve the accuracy of substance use measurement in behavioral and clinical HIV research, especially in contexts where stigma or criminalization may inhibit disclosure.

**Supplementary Information:**

The online version contains supplementary material available at 10.1007/s10461-025-04832-6.

## Resumen

Las investigaciones que relacionan el consumo de sustancias con conductas de riesgo para el VIH y con la adherencia a la terapia antirretroviral suelen basarse en datos autoinformados. Sin embargo, estudios en países de ingresos altos muestran que los hombres que tienen sexo con otros hombres (HSH) a menudo subreportan su consumo en comparación con pruebas biológicas objetivas. No se han realizado comparaciones similares entre HSH en África subsahariana. Comparamos datos autoinformados y pruebas objetivas de consumo de sustancias entre HSH que participaron en HPTN 075, un estudio de cohorte observacional, realizado en múltiples sitios en Kenia, Malaui y Sudáfrica. Se analizaron 734 muestras de orina de 382 participantes para detectar el metabolito del alcohol etil glucurónido y otras 43 sustancias. Estas pruebas identifican consumo de alcohol en las 48–72 horas previas, y de otras sustancias hasta 7 días antes, según el tipo de droga. De las 734 muestras, 159 (21.7%) fueron positivas para etil glucurónido. Para el 97,5% de esos casos había datos autoinformados; el consumo fue confirmado en 141 (91.0%) de ellos. Sesenta muestras (8.2%) resultaron positivas para al menos una de las 43 sustancias restantes. Había datos autoinformados para el 95.0% de esas muestras, pero solo en 19 (33.3%) se reconoció el consumo. Estos hallazgos sugieren que el consumo de alcohol se informa con mayor precisión que el de drogas, el cual probablemente se subreporta debido a su ilegalidad y estigma social. Se recomienda complementar los datos autoinformados con pruebas objetivas para mejorar la precisión en investigaciones conductuales y clínicas sobre el VIH, especialmente en contextos donde el estigma o la criminalización dificultan la divulgación.

## Introduction

Many studies have shown that substance use is positively associated with behaviors that increase the risk of HIV transmission, such as not using condoms, having multiple sexual partners and not adhering to the use of pre-exposure prophylaxis [1–8], and is negatively associated with adherence to antiretroviral treatment [9–12]. However, in most of these studies, the assessment of substance use was based on self-report [1], which may not always capture actual use [13]. Study participants may choose not to report substance use since it is stigmatized and because use may involve illicit substances; the decision to report substance use may also be impacted by social desirability bias [14, 15]. In addition, some people might not know what substances they used or may not remember their substance use. These factors may lead to under-reporting of substance use which could impact assessments of the association between substance use and other behaviors. Further studies are needed to understand the accuracy of self-reported substance use.

Previous research compared outcomes of biospecimen testing with self-reported substance use among men who have sex with men (MSM) in the United States. For example, White and colleagues [16] found that Black MSM were less likely to report recent use of marijuana and cocaine than White MSM, but were equally as likely to test positive for either substance, after adjusting for age, education, income, sexual orientation, and history of arrest. Similar studies using laboratory measures to assess the accuracy of self-reported substance use have not yet been conducted among MSM in Africa. This is a critical gap since substance use is a growing problem in Africa [17–20]. In a systematic review, the 12-month prevalence of alcohol use disorder and alcohol dependence among African adults was estimated to be 9.5% and 4.3%, respectively [21]. Self-report of substances may further be affected by the low acceptance of same-sex sexuality in Africa. Although there is variety between countries [22, 23], attitudes in Africa towards same-sex sexuality are overwhelmingly negative [24].

In this study, we evaluated whether participants whose urine samples tested positive for alcohol and other substances reported use of these substances. Data for this study were collected in the HIV Prevention Trials Network (HPTN) 075 study, which assessed the feasibility of recruiting and retaining MSM at four study sites in Africa [25].

## Methods

After having discussed the risks, benefits and aims of the study, participants provided informed consent for participation in HPTN 075. Study procedures were approved by local and international institutional review boards, including Johns Hopkins University and College of Medicine Ethics Research Committee (COMREC) for the Malawi site; Scientific Ethics Review Unit (SERU) for the Kenya site; University of the Witwatersrand for the Soweto site; the University of Cape Town for the Cape Town site; and the New York State Psychiatric Institute. Consent for the storage and use of left over urine samples was obtained independently from participation in the study overall.

HPTN 075 was a prospective cohort study conducted between 2015 and 2017 at one site in Kenya and Malawi, and two sites in South Africa [25]. The study enrolled 401 persons assigned male sex at birth, between 18 and 44 years of age, who reported anal intercourse with a man in the past 3 months. People were eligible to enroll regardless of HIV status; the number of participants living with HIV was capped at 20 per site. Persons with HIV who reported already being in HIV care or taking antiretroviral (ARV) drugs were excluded to be able to assess engagement in HIV care. Study participation included in-person biobehavioral assessments at screening and at five subsequent study visits over a one-year period. Interviews were conducted in-person by research staff specifically trained to administer the survey. In addition to the survey interviews, all study visits included HIV risk reduction counselling, assessment of social impacts, medical examinations, collection of blood samples, and HIV testing (if participants had tested HIV negative at the prior visit); urine samples were collected at the enrollment visit (visit 1) and at the final, 52-week follow-up visit (visit 5). Self-reported data on drug and alcohol use and urine samples used for this study were collected at the same study visits. Additional information for the HPTN 075 study is reported elsewhere [25].

### Self-report of substance use

#### Alcohol use

Alcohol consumption was assessed using responses to the first item of the AUDIT-C questionnaire [26, 27], which asks how often participants have a drink containing alcohol; the response options include: never; monthly or less; two to four times a month; two to three times a week; and four or more times a week. No timeframe was specified. The same question was asked at enrollment and at the final study visit. Participants who reported any alcohol use, regardless of frequency, were categorized as having used alcohol.

#### Other substance use

Questions about use of other substances were introduced with the following instruction: “The next questions are about other substances you could have used, including substances prescribed by a doctor (like pain medications) that you might have taken for reasons or in doses other than prescribed, and recreational or illegal drugs. When referring to these substances, please include any of the following: cannabis (marijuana, dagga, bhang, ganja, puga, pot, grass, hash, etc.); nyaope (whoonga or wunga; ARV mixed with other drugs); inhalants (turpentine, nitrous oxide, shoe glue, glue, petrol, gas, paint thinner, etc.); methamphetamine (tik, speed, crystal meth, ice, etc.); sedatives or sleeping pills (mandrax, Valium, Serepax, Ativan, Xanax, Librium, Rohypnol, GHB, etc.); cocaine (coke, crack, etc.); Street opioids (heroin, opium, etc.); prescription opioids (fentanyl, oxycodone [OxyContin, Percocet], hydrocodone [Vicodin], methadone, buprenorphine, etc.); and prescription stimulants (Ritalin).” After this introduction, two questions were asked: “In the past year/past 6 months, how often have you used prescription drugs for non-medical reasons?” and “In the past year/past 6 months, how often have you used recreational drugs?” A timeframe of one year was used at the enrollment visit and a timeframe of 6 months was used at the final study visit. Response options were: never; once or twice monthly; once or twice weekly; once or twice daily; and almost daily. Participants who reported any use of these substances were categorized as having used substances other than alcohol.

### Urine testing

Urine was tested for 44 analytes, including one biomarker for alcohol exposure (ethyl glucuronide) and 43 biomarkers for prescription and illicit use of other substances. Testing was performed using liquid chromatography-tandem mass spectrometry (see Appendix). Positive tests for alcohol exposure indicate that alcoholic drinks had been used in the previous 48 to 72 h. Positive tests for one or more of the other 43 substances indicate use up to 7 days prior, with the exact window varying by substance.

### Analysis

Sample characteristics were calculated using means and percentages. Correspondence between urine test results and self-reported substance use were explored using cross tabulation. Correlates of personal characteristics with positive urine tests indicating substance use and self-reported substance use were identified using Chi square analysis. Adjusted standardized residuals were inspected to determine which cells had a value equal to or greater than |1.96|, indicating counts that differed significantly from expected values.

## Results

Urine samples were available from 382 participants at the enrollment visit (19 participants opted out of urine testing). The median age of the 382 participants was 23 years (range 18 − 14). At enrollment, 18.4% of the participants had tested positive for HIV; 17 participants seroconverted during the study. Additional personal characteristics are reported in Table 1. Three hundred fifty-two (92%) of the 382 participants also provided urine samples at the final visit. The analysis in this report included a total of 734 samples from 382 participants. Of all participants who provided urine samples, 126 (33.0%) tested positive for alcohol and 50 (13.1%) tested positive for drugs (at the first visit, at the last visit, or at both visits). The samples of 15 (3.9%) participants tested positive for both alcohol and drug use at the same study visit, with two of them testing positive for both alcohol and drug use at both study visits.

**Table 1:** Demographic and psychosocial characteristics of participants in HPTN 075 with urine samples (N=382)

	N/mean^1^	%/SD
Site		
Cape Town, South Africa	86	22.5
Kisumu, Kenya	96	25.1
Blantyre, Malawi	100	26.2
Soweto, South Africa	100	26.2
Age (years)	24.4	5.53
Education level		
Less than grade 12	135	35.4
Grade 12 completed	161	42.3
College/university	85	22.3
Employment status		
Employed	187	49.6
Unemployed	167	44.3
Disability/other	23	6.1
Sexual attraction		
Only men	179	47.2
Men and women or women only	200	52.8
Sexual identity		
Gay	238	62.8
Bisexual or heterosexual	141	37.2
In intimate sexual relationship with a man		
No	72	19.3
Yes	302	80.7
HIV status at enrollment		
HIV-Positive	70	18.4
HIV-Negative	311	81.6
Seroconverted during study		
No	294	94.5
Yes	17	5.5

^1^Ns may not equal the total sample due to missing data.

### Alcohol tests

Of the 734 urine samples tested for ethyl glucuronide, 159 (21.7%) were positive, indicating that alcohol had been consumed in the previous 48 to 72 h. Positive tests were obtained for 126 (33.0%) participants: 33 participants tested positive on both study visits and 93 participants on one visit only.

Table 2 provides the associations of several personal characteristics with positive test outcomes. Detection of alcohol was significantly associated with study site (Chi square = 10.41, *p* = .015): compared with the other three sites, alcohol was more likely to be detected among participants in Blantyre. Participants with positive test results were likely to be older compared to those who tested negative (25.7 versus 24.0 years; *t* = -2.04, *p* = .042). Detection of alcohol was also associated with employment status and HIV status at enrolment. Alcohol was more likely to be detected among participants who were employed and less likely among participants who were unemployed (including students) (Chi square = 7.21, *p* = .027). Finally, alcohol was more likely to be detected among participants who tested HIV positive at the start of the study compared to those who tested HIV negative (Chi square = 4.87, *p* = .027); participants who seroconverted during the study were not more likely to have alcohol detected than those who continued to test HIV negative (Chi square = 0.42, *p* = .518).

**Table 2:** Alcohol detection and self-reported use among participants in HPTN 075 with urine samples (N=382)

	Alcohol detected	Alcohol use not reported						
	n/N	%	Chi square	*p*	n/N	%	Chi square	*p*
Site			10.41	.015			9.03^1^	.018
Cape Town, South Africa	24/86	27.9			0/22	0.0		
Kisumu, Kenya	27/96	28.1			2/27	7.4		
Blantyre, Malawi	46/100	46.0			10/46	21.7		
Soweto, South Africa	29/100	29.0			1/29	3.4		
Education level			0.66	.720			3.25^1^	.190
Less than grade 12	48/135	35.6			8/47	17.0		
Grade 12 completed	52/161	32.3			4/51	7.8		
College/university	26/85	30.6			1/26	3.8		
Employment status			7.21	.027			1.23^1^	.598
Employed	71/187	38.0			6/71	8.5		
Unemployed	43/167	25.7			6/41	14.6		
Disability/other	10/23	43.5			1/10	10.0		
Sexual attraction			0.44	.506			2.75	.097
Only men	56/179	31.3			3/55	5.5		
Men and women or women only	69/200	34.5			10/68	14.7		
Sexual identity			0.04	.843			0.51	.475
Gay	80/238	33.6			7/78	9.0		
Bisexual or heterosexual	46/141	32.6			6/46	13.0		
In intimate relationship with a man			0.22	.639			1.08	.299
No	22/72	30.6			12/99	12.1		
Yes	101/302	33.4			1/22	4.5		
HIV status at enrollment			4.87	.027			0.01	.921
HIV-Positive	31/70	44.3			3/30	10.0		
HIV-Negative	95/311	30.5			10/94	10.6		
Seroconverted during study			0.42	.518			0.91	.341
No	91/294	31.0			9/90	10.0		
Yes	4/17	23.5			1/4	25.0		

Ns may not equal the total sample due to missing data.

^1^Fisher’s exact tests were calculated due to the number of cells with an expected frequency of 0.

### Tests for other substances

Positive test results were obtained for 12 of the 43 analytes, including tetrahydrocannabinol carboxylic acid (THC_COOH) (*n* = 17), benzoylecgonine (*n* = 14), methamphetamine (*n* = 12), amphetamine (*n* = 9), cis-tramadol (*n* = 4), cocaine (*n* = 3), methylene dioxyamphetamine (MDA) (*n* = 3), codeine (*n* = 2), flurazepam (*n* = 2), oxymorphone (*n* = 2), dihydrocodeine (*n* = 1), and methylphenidate (*n* = 1). No positive results were obtained for the following 31 analytes: 6-monacetylmorphine, α-hydroxyalprazolam, alprazolam, buprenorphine, carisoprodol, chlordiazepoxide, clonazepam, diazepam, 2-ethylidene-1,5-dimethyl-3,3-diphenylpyrrolidine (EDDP), fentanyl, hydrocodone, hydromorphone, lorazepam, methyldiethanolamine (MDEA), 3,4-methylenedioxymethamphetamine (MDMA), meperidine, methadone, morphine, naloxone, nitazene, norbuprenorphine, nordiazepam, norfentanyl, noroxycodone, oxazepam, oxycodone, phentermine, tapentadol, temazepam, xylazine, and zolpidem.

Analytes indicating use of one or more of the 43 substances were detected in 60 (8.2%) of the 734 samples indicating use of these substances up to 7 days prior, with the exact window varying by substance. One sample had positive results for three analytes; eight samples had positive results for two analytes, and 51 samples had a positive result for one analyte. The 60 positive test results were obtained from the samples of 50 participants: 10 participants had positive results in samples from both visits and 40 had positive results from a sample from one visit only.

Table 3 provides the associations of several personal characteristics with positive test outcomes for drug use. Detection of drugs was also associated with study site (Chi square = 17.88, *p* <.001), with detection more likely among participants from Cape Town, and less likely among participants from Blantyre, compared to participants from the two other study sites. The association with age was marginally significant (*t* = -1.83, *p* = .073); the mean age of participants with positive test results was 26.0 years and those with negative results was 24.2 years. Positive drug tests were more likely among participants who were not in a steady intimate same-sex relationship (Chi square = 6.52, *p* = .011) and among participants who tested HIV positive at the start of the study (Chi square = 9.37, *p* = .002); participants who seroconverted during the study were not more likely to have alcohol detected than those who continued to test HIV negative (Chi square = 2.14, *p* = .144).

**Table 3:** Drug detection and self-reported use among participants in HPTN 075 with urine samples (N=382)

	Drugs detected				Drug use not reported			
	n/N	%	Chi square	*p*	n/N	%	Chi square	*p*
Site			17.88	<.001			2.55^1^	.476
Cape Town, South Africa	22/86	25.6			16/22	72.7		
Kisumu, Kenya	5/96	5.2			3/4	75.0		
Blantyre, Malawi	13/100	13.0			12/13	92.3		
Soweto, South Africa	10/100	10.0			7/10	70.0		
Education level			0.43	.808			0.93^1^	.745
Less than grade 12	19/135	14.1			14/19	73.7		
Grade 12 completed	19/161	11.8			16/19	84.2		
College/university	12/85	14.1			8/11	72.7		
Employment status			1.10	.579			5.22^1^	.045
Employed	21/187	11.2			14/20	70.0		
Unemployed	25/167	15.0			22/25	88.0		
Disability/other	3/23	13.0			1/3	33.3		
Sexual attraction			0.77	.381			0.00	.977
Only men	26/179	14.5			20/26	76.9		
Men and women or women only	23/200	11.5			17/22	77.3		
Sexual identity			0.02	.900			0.27	.606
Gay	31/238	13.0			24/30	80.0		
Bisexual or heterosexual	19/141	13.5			14/19	73.7		
In intimate relationship with a man			6.52	.011			2.89	.089
No	16/72	22.2			10/16	62.5		
Yes	33/302	10.9			27/32	84.4		
HIV status at enrollment			9.37	.002			0.35	.557
HIV-Positive	17/70	24.3			14/17	82.4		
HIV-Negative	33/311	10.6			24/32	75.0		
Seroconverted during study			2.14	.144			N.A.	
No	33/294	11.2			24/32	75.0		
Yes	0/17	5.6			0/0	0.0		

Ns may not equal the total sample due to missing data.

^1^Fisher’s exact tests were calculated due to the number of cells with expected frequency of 0.

### Reporting of alcohol use among participants with positive test results

In total, participants reported any alcohol use at 141 (91.0%) of the 155 visits corresponding to the positive samples (self-reported data were missing for four samples) (Table 4). Of the 33 participants with positive urine test results for alcohol on both visits, 27 participants reported using alcohol on both visits; three reported using alcohol on only one of the two visits; two participants reported using alcohol on one visit, but their self-report was missing on the other visit; and one person did not report alcohol use on either visit. Of the 93 participants with a positive test result on one visit only, 82 reported using alcohol on the associated visit (self-report was missing for two cases). In summary, while almost all participants with positive alcohol test results reported using alcohol, 13 (10.5%) out of 124 participants reported never to use any alcohol at either one or both occasions (two participants were missing).

**Table 4:** Self-reported alcohol use and use of other substances by number of tests performed among participants with positive urine test results

	Number of positive tests	Reported use	Reported not to use	Self-report missing	Number of participants
**Alcohol**					
*Positive test at both visits*					
Reported use at both visits	54	54	0	0	27
Reported use at one of two visits	6	3	3	0	3
Reported use at one visit, self-report missing at other visit	4	2	0	2	2
Reported at either visit not to use	2	0	2	0	1
*Positive test at one visit*	93	82	9	2	93
Total	159	141	14	4	126

**Other substances**					
*Positive test at both visits*					
Reported use at both visits	10	10	0	0	5
Reported use at one of two visits	6	3	3	0	3
Reported not using at one visit, self-report missing at other visit	2	0	1	1	1
Self-report missing at both visits	2	0	0	2	1
*Positive test at one visit*	40	6	34	0	40
Total	60	19	38	3	50

Among those with a positive test for alcohol use, none of the personal characteristics, except for study site, were associated with not reporting alcohol use while having a positive urine test (Table 2). Participants in Blantyre were more likely to report never using alcohol compared to participants from the three other study sites (Chi square = 9.03, *p* = .018).

### Reporting of substance use among participants with positive test results

In total, drug use in the preceding year or six months was reported at 19 (33.3%) of the 57 visits corresponding to the samples with one or more drugs detected (self-report was missing for three samples) (Table 4). Of the ten participants with positive urine test results at both visits, five reported drug use on both visits; three reported drug use on one of the two visits but not on the other visit; one did not report drug use at one visit and self-report of drug use was missing for the other visit; and self-report was missing for both visits for the final person. Of the 40 participants with positive test results on one visit, six reported having used any drugs in the preceding year or six months. In summary, drug use was more frequently not reported than alcohol use: 38 (77.6%) of the 49 participants who tested positive for drug use did not report having used any drugs (one participant was missing).

Among the participants with positive urine test results for drug use, those who reported not having used any drugs were significantly younger than those who did (31.8 and 24.0 years; *t* = -2.69, *p* = .020). Compared with participants who were employed, those who were unemployed were significantly more likely and those with a disability or another employment status were significantly less likely not to have reported having used any drugs (Fisher’s exact test = 5.22, *p* = .045); the number of participants with disability or another employment status is very small, though. No other characteristics were associated with not reporting any use of drugs (Table 3).

## Discussion

This report compared the results of substance use testing of 734 urine samples from 382 African MSM with their self-reported use. Recent alcohol use, in the previous 48 to 72 h, was detected in a little over one in five samples, and the recent use of twelve other substances (up to 7 days prior to the test) in almost one out of ten samples. The proportion of positive urine samples varied per study site and was higher among participants who tested HIV positive at enrollment. Most participants with positive samples, 76.7%, reported using alcohol. Drug use (in the preceding year or preceding six months, dependent upon study visit) was only reported by participants in 9.0% of the cases with positive urine tests indicating recent use.

These findings regarding correspondence between objective urine test results and self-reported substance use are consistent with reports of studies conducted in high-income countries. For example, among electronic dance music party attendees in New York City, 51.1% of participants reported cocaine use while 80.0% had positive hair test results for cocaine [28]. In a sample of 500 individuals from a one-year smoking cessation clinical trial, Clark and colleagues [13] found that almost two-thirds of individuals who tested positive for one or more of a series of substances, did not report use when assessed in interviews, with non-concordance increasing over time. In a systematic review of 28 studies among adolescents and youth up to 26 years, Folk et al. [29] concluded that agreement between self-reported data and laboratory-documented substance use was low to moderate. Some studies that included participants who had not used substances, based on both self-report and testing of biospecimens, reported high concordance, although the validity of self-report of non-use was greater than that of self-report of use [30, 31].

Underreporting is not limited to substance use. Notably, among participants in HPTN 075 who were screened in interviews for being in HIV treatment, 36 denied having been diagnosed with HIV, while objective tests showed the presence of antiretroviral (ARV) drugs, indicating that they were on antiretroviral treatment [32]. In the context of HIV, studies have also documented the overreporting of specific behaviors, in particular the use pre-exposure prophylaxis (PrEP). Hebel and colleagues [33] found among 3987patients who self-reported being adherent to PrEP, that 14% demonstrated recent nonadherence when tested with a liquid chromatography–mass spectrometry test. Baker and colleagues [34] concluded in a study among 159 young MSM that overreporting of adherence, as compared with dried blood spot test results, was fairly common, occurring in 40% of their participants. They identified only a few participants who underreported their adherence. Amico and colleagues [35] found a similar rate of overreporting among participants in the iPrEx open-label extension: about 17% of 985 participants who reported dosing of PrEP in the past 3-day did have quantifiable levels of drug. Surprisingly, among those who reported no recent doses, 18% of 187 participants had quantifiable levels of drug, suggesting that accounts of PrEP use can be significantly overreported as well as underreported.

The higher level of correspondence in our study for alcohol use compared to use of other substances could reflect a higher prevalence of alcohol use in the population in general [36]. Alcohol use is also legal and less stigmatized than drug use, which could impact self-reporting. Clark and colleagues [13] showed that use of substances with more stigma was associated with higher rates of false negative reports compared to less stigmatized substances. In a review of studies Zimmerman and colleagues [37] also found more frequent underreporting for drugs that were used less frequently or that are associated with greater legal penalties.

More frequent use and less stigmatization of specific substances might facilitate self-report, but it does not explain why some people are more likely to report correctly than others. Other studies have identified several other correlates of lack of correspondence between test results and self-reported data, including lower cognitive functioning, an external locus of control, younger age, and lower educational attainment [13, 35, 38]. In addition to age, we only found study site and employment status to be associated with underreporting. These factors also do not explain why some participants provide correct self-report, while others do not. Consequently, we can only speculate about the underlying processes that explain the lack of agreement observed in our study.

Stigma about substance use or concern about potential legal consequences of reporting one’s use may have prohibited some participants in our study from accurately reporting their use. Some participants’ self-report may also have been impacted by social desirability bias [14, 15] and a wish to present oneself in a positive light. In addition, some participants might not have known that they used specific substances. Methodological factors, related to the process of asking questions, retrieving information, and responding to questions, may have played a role as well [39]. For instance, it is quite possible that the specific questions about drug use that were used in this study did not resonate with the participants’ actual drug use practices. Although the wording of the questions included a list of local drug names, the questions were not based on an understanding of the local culture of drug use. As a consequence, participants might not have understood the questions as intended and might not have made a connection with their drug use practices. This would suggest that one of the ways to improve the quality of reporting would be to develop survey questions that are grounded in local drug use practices.

### Limitations

One limitation of this study is that the timeframe used for self-reported data collection exceeded the detection window for the presence of substances in the urine samples; some of the self-reported substance use is likely to have occurred outside of that window, potentially inflating the observed agreement. In addition, the different timeframes used for self-reported data collection and laboratory assessments made it impossible to assess whether there were any false negative test results. Finally, there was limited power to explore correlates of lack of agreement between self-report data and data from urine testing.

### Conclusion

Including laboratory testing improves the accuracy for assessing alcohol use among African MSM; this difference was more striking when assessing use of other substances. In settings where this type of testing is not feasible or affordable, assessment of substance use based on self-report should be done in a way that promotes accurate recollection and avoids bias, preferably using validated measures or other strategies to promote valid responses [29, 40]. When interpreting self-reported substance use data and its associations, under-reporting should be considered.

## Electronic Supplementary Material

Below is the link to the electronic supplementary material.

Supplementary Material 1.
