# Supplementary material for: Comparison of Self-Reported Substance Use with Outcomes of Urine Testing among Men Who Have Sex with Men in Africa Participating in HPTN 075
Source: AIDS Behav. 2025 Sep 13;29(12):3960–9. doi: 10.1007/s10461-025-04832-6 (PMC12435900; doi:10.1007/s10461-025-04832-6)
Supplement: Supplementary file 1 — Supplementary Material 1. [file 10461_2025_4832_MOESM1_ESM.docx]

**Online Resource 1: Toxicology Testing Methodology**

**Supplementary file to “Comparison of self-reported substance use with outcomes of urine testing among men who have sex with men in Africa participating in HPTN 075”**

Theodorus G. M. Sandfort^1^, Susan H. Eshleman^2^, Justin Knox^1,3^, Autumn Breaud^2^, Katie Weaver^2^, Emily Kerubo^4^, Ravindre Panchia^5^, erica l. hamilton^6^, Vanessa Cummings^2^, Bill Clarke^2^

^1^ HIV Center for Clinical and Behavioral Studies, New York State Psychiatric Institute and Columbia University, New York, NY, USA

^2^ Department of Pathology, Johns Hopkins University School of Medicine, Baltimore, MD, USA

^3^ Department of Sociomedical Sciences, Columbia University, New York, NY, USA

^4^ Kenya Medical Research Institute (KEMRI) CDC, Kisumu, Kenya

^5^ Perinatal HIV Research Unit, University of the Witwatersrand, Soweto, South Africa

^6^ Science Facilitation Department, FHI 360, Durham, NC, USA

Corresponding author: Theodorus G. M. Sandfort (tgs2001@cumc.columbia.edu)

**Toxicology Testing Methodology**

The validated method is a targeted liquid chromatography/high resolution mass spectrometry (LC/HRMS) qualitative assay based on data dependent acquisition (DDA) and library matching to be used for qualitative screening for drugs of abuse in urine. The purpose of the assay is to detect exposure to a drug (or drugs) within the detection window for the analytes of interest.

For most drugs in the assay, a positive result is based on a peak area consistent with or higher than the lower limit of detection (LOD) concentration and a library matching score of > 75. The exceptions are 6-monoacetylmorphine (6-MAM), buprenorphine, buprenorphine-glucuronide, norbuprenorphine, tetrahydrocannabinol carboxylic acid (TCH-COOH), ethylglucuronide, and hydroxyalprazolam, for which a positive result is based on retention time, precursor exact mass within 10 parts per million (ppm) of the expected mass, peak area consistent with or higher than the LOD concentration, and manual review confirmation – these seven compounds will always require manual review.

The Library Score for matching of experimental and reference spectra is determined solely from the MS2 fragmentation spectrum. The Library Matching (such as that used by NIST, Thermo, Sciex, and others) does not explicitly use ion ratios for the MS2 fragments but works by reducing the MS2 spectrum to a dot product multidimensional vector; each MS2 fragment peak is an array of m/z and intensity (this vector is affiliated with the precursor ion, but the precursor ion does not contribute to the vector). As such, all detected fragments contribute to the library score. This process is applied to both experimental spectra and reference spectra; there is one vector for a library hit (reference MS2 spectrum) and one vector for the query (experimental MS2 spectrum), and the score is based on the cosine of the angle between them. Since each vector is constructed with the real measured values, the vectors incorporate the continuous variations in mass and intensity. This is how one can observe nearly identical visual spectra, and yet not obtain a perfect Dot Product/Library score – the accumulated small differences in mass and intensity add up to a deviation in the resulting vectors. Vector contributions are based on mass-to-charge ratio (m/z) and abundance contribution; the algorithms for this can be differentially weighted for one of these factors or the other, or equally balanced.

The library matching algorithm used in the current method is called “HighChem” or “Dot Product” (full name is HighChem Optimized Dot Product). There is no additional weighting for m/z or intensity, but it does have a penalty for very sparse spectra. It is harder to get a very high score with a very sparse spectrum unless the m/z:intensities are near identical – giving rise to nearly identical vectors.

*Verification of Assay LOD Reporting Cutoff*

Verification of the reporting cutoff concentration involved analysis of 10 replicates of each analyte in urine at the proposed limit of detection for the analyte, at 50% less than the LOD, and 50% greater than the LOD.

At each concentration, the mean, standard deviation of the mean (SD), and % coefficient of variation (CV) was calculated to assess signal (peak area) precision for the assay. In addition, the signal range of the mean +/- 2 SD was calculated and assessed for overlap of measured peak area distribution for each concentration to verify the assay cutoff.

Precision: the % CV for each LOD tested did exceed 20% for automated resulting; for those analytes subject to manual review, a % CV of <20% was considered desirable, but not required.

Cutoff Verification: For the cutoff concentration to be considered verified, the range of the mean +/- 2 SD for the cutoff concentration and +/- 50% x LOD must not exhibit overlap greater than 20%. The verified cutoff concentration can be seen in Table A.

**Table A: Verified LOD Cutoff Concentrations**

| **Analyte** | **LOD (ng/mL)** |
| --- | --- |
| 6-monacetylmorphine | 25 |
| α-hydroxyalprazolam | 50 |
| Alprazolam | 50 |
| Amphetamine | 100 |
| Benzoylecgonine | 15 |
| Bup-Glucuronide | 50 |
| Carisoprodol | 100 |
| Chlordiazepoxide | 15 |
| Cis-Tramadol | 15 |
| Clonazepam | 50 |
| Cocaine | 15 |
| Codeine | 100 |
| Diazepam | 50 |
| Dihydrocodeine | 25 |
| EDDP | 100 |
| Ethyl Glucuronide | 200 |
| Fentanyl | 5 |
| Flurazepam | 15 |
| Hydrocodone | 100 |
| Hydromorphone | 100 |
| Lorazepam | 50 |
| MDA | 50 |
| MDEA | 100 |
| MDMA | 50 |
| Meperidine | 100 |
| Methadone | 100 |
| Methamphetamine | 15 |
| Methylphenidate | 5 |
| Morphine | 100 |
| Naloxone | 25 |
| Nitazene | 5 |
| Norbuprenorphine | 15 |
| Nordiazepam | 25 |
| Norfentanyl | 15 |
| Noroxycodone | 50 |
| Oxazepam | 15 |
| Oxycodone | 100 |
| Oxymorphone | 50 |
| Phentermine | 100 |
| Tapentadol | 50 |
| Temazepam | 15 |
| THC-COOH | 100 |
| Xylazine | 5 |
| Zolpidem | 5 |
